# Multi-Locus Integration of Antimicrobial Peptide Api137 in *Saccharomyces cerevisiae* Based on Ty Transposons: Expression and Activity Analysis

**DOI:** 10.3390/microorganisms14081728

**Published:** 2026-08-06

**Authors:** Ruiqian Wang, Jia Song, Bo Sun, Meiling Zhang, Kuanbo Liu, Xue Yan, Ruimin Li, Wanzhong Zhang, Chen Zhao

**Affiliations:** 1 College of Chemical Engineering, Shenyang University of Chemical Technology, Shenyang 110142, China; 2 Key Laboratory of Grain and Oil Biotechnology, Academy of National Food and Strategic Reserves Administration, Beijing 100037, China; 3 Key Laboratory of Feed and Livestock and Poultry Products Quality & Safety Control, Ministry of Agriculture and Rural Affairs, New Hope Liuhe Co., Ltd., Chengdu 610023, China; 4 College of Environmental and Safety Engineering, Shenyang University of Chemical Technology, Shenyang 110142, China

**Keywords:** antimicrobial peptide, *Saccharomyces cerevisiae* CENPK2, heterologous expression, Ty transposon, activity detection

## Abstract

Antimicrobial peptides (AMPs) are promising alternatives to antibiotics for combating multidrug-resistant bacteria, yet their practical application is hindered by the low content of natural AMPs and the high cost of chemical synthesis. In this study, we developed a high-efficiency heterologous expression system for the proline-rich cationic antimicrobial peptide Api137 in *Saccharomyces cerevisiae* CENPK2 by engineering the Ty retrotransposon system composed of multi-locus integration. Recombinant plasmids carrying Api137 encoding elements were constructed and integrated into the CENPK2 genome, generating the engineered strain CENPK2 + Ty1-2/2/3/4. The target fusion peptide (5.2 kDa) was successfully expressed and identified by Tris-tricine-SDS-PAGE and liquid chromatography–tandem mass spectrometry (LC-MS/MS). The quantification of Api137 from fermentation broth was applied by high-performance liquid chromatography (HPLC) which showed that the yield of tandem peptide in the fermentation supernatant reached 20.5 mg/L and the intracellular retention rate was 33.0%. Comparative analysis of MIC and MBC values revealed that the biologically synthesized Api137 exhibited slightly superior antibacterial activity relative to the chemically synthesized Api137. In vitro functional assays demonstrated that the fermentation supernatant of the engineered strain exhibited broad-spectrum antibacterial activity against five pathogenic bacteria, with a maximum antibacterial rate of 92.9% against *Aeromonas veronii*. Hemolysis assays and cytotoxicity tests confirmed that the fermentation supernatant exhibited neither hemolytic activity nor cytotoxicity. Moreover, the expression of Api137 did not impose a metabolic burden on the host. This study establishes a Ty transposon-mediated strategy for the high-level expression of Api137 in *S. cerevisiae*, which significantly demonstrates the antibacterial activity of Api137 while ensuring excellent biosafety.

## 1. Introduction

In recent years, antimicrobial peptides (AMPs) have garnered considerable attention for their broad-spectrum and potent antibacterial activity as well as their crucial role in innate immunity [1,2]. However, natural AMPs face several research bottlenecks, including low abundance in natural organisms, poor stability, and difficulties in extraction and purification [3]. Additionally, chemically synthesized AMPs are associated with high costs and inconsistent bioactivity, which restrict their large-scale production and practical application. Api137 (ID: GNNRPVYIPQPRPPHPRL) is a proline-rich cationic antimicrobial peptide, presumed to be an insect-derived AMP analog (the name is derived from antimicrobial peptide from insect), and the number 137 represents its sequence-optimized version or screening batch. It is a potential candidate drug against multidrug-resistant bacteria [4]. Api137 exerts its antibacterial effect by specifically interfering with the assembly of the bacterial 50S ribosomal subunit and inducing the misfolding of precursor particles [5]. Meanwhile, it enters bacteria through the SbmA transporter and inhibits protein translation by binding to the ribosomal peptide exit tunnel [6].

*Saccharomyces cerevisiae* is a vital biological platform for bioconversion and the first eukaryotic model organism with a fully sequenced genome [7]. Its excellent fermentation efficiency and genetic stability make it one of the most important strains for industrial fermentation and basic research [8]. It possesses numerous advantages including rapid growth, simple operation, high safety, strong genetic manipulability, and superior fermentation capacity [9]. Therefore, improving its fermentation performance via gene editing has become a research hotspot at present. Ty sites in *S. cerevisiae* are LTR retrotransposons [10], belonging to the “copy-and-paste” transposable elements in eukaryotes. Laboratory strains such as *S. cerevisiae* CENPK2 harbor significantly more Ty copies than other strains [11]. Each intact Ty element (5.4–5.9 kb, approximately 6 kb) carries the sequences required for its expression and transposition which from six LTR retrotransposon families, four of which are active (Ty1, Ty2, Ty3, and Ty4) [12], and there are different subfamilies such as Ty1′ and Ty1/2 which are of great value for studying genome evolution and developing gene-editing tools [13]. Qi et al. [14] found that all Ty1 elements are potential targets for CRISPR/Cas9 and characterized the nature of diploid genomic changes with detectable allelic and non-allelic recombination. The expression of CRISPR/Cas9 targeting Ty1 elements is an efficient method to generate a variety of novel chromosomal rearrangements.

To address the challenges of high cost in chemical synthesis, as well as low expression level and unstable biological activity of Api137 in organisms, this study employed gene editing and multi-locus modification strategies [3] to construct a high-expression strain of the Api137 antimicrobial peptide by optimizing the Ty transposon system in *S. cerevisiae*, so as to improve the expression level and stable activity of the antimicrobial peptide in organisms. Furthermore, the reconstruction of Ty loci enhanced the antibacterial activity of the antimicrobial peptide produced by the fermentation of the novel *S. cerevisiae* strain. This study provides a feasible, low-cost strategy for the practical application of high-activity AMPs. It also offers a promising alternative to conventional antibacterial agents and lays a foundation for further research and translational application of AMPs.

## 2. Materials and Methods

### 2.1. Strains and Reagents

The strains used for antimicrobial activity determination, *Escherichia coli* ATCC K88, *Salmonella Typhimurium* ATCC 9027, *Staphylococcus aureus* CICC 21664, *Aeromonas veronii* ATCC 13124, and *Listeria monocytogenes* ATCC 19114, were preserved in our institute. Restriction endonucleases were obtained from New England Biolabs (NEB, Ipswich, MA, USA) and the primers used are detailed in Table 1. A plasmid extraction kit was obtained from Genstar (Beijing, China). All other chemical reagents were of analytical grade. *E. coli* Top10 was used for plasmid construction and *S. cerevisiae* CENPK2 was used for recombinant protein expression. The antimicrobial peptide Api137 was chemically synthesized via solid-phase synthesis by Sangon Biotech (Shanghai, China). The purity of all peptides was above 95%.

### 2.2. Construction of Strains and Plasmids

In this study, the recombinant plasmids pUC57-Ty2(KILEU2)-TEF1-alpha57-70-Api137x2-Flag (stored in our laboratory) were used as the amplification template. Notably, the Flag tag as a flexible linker embedded in this plasmid was designed to serve two copies of the Api137 gene in tandem, ensuring stable spatial connection and correct expression of the dual Api137 fragments without disrupting their structural integrity. The target gene fragment containing the tandem Api137 repeats and Flag tag was amplified by PCR with KOD high-fidelity DNA polymerase and the primer pair TEF1-F/Api137-1′-R. The plasmid pUC57-Ty1-2-KIURA3-CL1 was double-digested with *Eco*RI and *Sph*I, and the linearized carrier and target amplified fragment were ligated via Gibson assembly. The ligation product was chemically transformed into *E. coli* Top10 competent cells, and positive transformants were identified by colony PCR. Plasmids were isolated from verified positive clones using a plasmid miniprep kit, and the plasmids were submitted to Sangon Biotech for Sanger sequencing, generating pUC57-Ty1-2-KIURA3-Api137x2-FLAG (Ty1-2). Using the constructed Ty1-2 plasmid as the template, the target fragment carrying the tandem Api137 genes was re-amplified by PCR with KOD high-fidelity DNA polymerase and the primer pair TEF1-F/Api137-2′-R. The backbone plasmids pUC57-Ty2-KILEU2-CL1, pUC57-Ty3-ScHIS3-CL1, and pUC57-Ty4-ScTRP1-CL1 were individually double-digested with *Eco*RI and *Sph*I. Three recombinant plasmids harboring the identical Flag tag-tethered dual Api137 expression cassette, pUC57-Ty2-KILEU2-Api137x2-FLAG(Ty2), pUC57-Ty3-ScHIS3-Api137x2-FLAG(Ty3) and pUC57-Ty4-ScTRP1-Api137x2-FLAG(Ty4), were constructed via Gibson assembly.

### 2.3. Construction of Recombinant CENPK2

Four recombinant plasmids were linearized by PCR using primers M13-M4 and M13-RV. The PCR products were blended with CENPK2 competent yeast cells for electroporation, then the mixture was evenly spread onto SC-Ura-Leu-His-Trp synthetic dropout selection plates, followed by incubation at 29 °C for 3–5 days. Single colonies were picked from the dropout plates. Genomic DNA extracted from lysis was used as the template for PCR identification, with primers CYC1-seq and ADH1-seq. Recombinant strains which showed correct PCR amplification profiles and grew stably on dropout medium were designated as CENPK2 + Ty1-2/2/3/4 (R-CENPK2).

Using the aforementioned electroporation protocol, two-combination and three-combination recombinant CENPK2 strains were constructed and designated as CENPK2 + Ty1-2/2 and CENPK2 + Ty1-2/2/3, respectively.

### 2.4. Flask Fermentation of Recombinant CENPK2

Single colonies of the identified recombinant CENPK2 were inoculated into YPD liquid medium and incubated at 29 °C at 220 rpm for 18 h. The seed cultures were subsequently transferred into 300 mL flasks containing 40 mL of YPD liquid medium at a 1% inoculation ratio, followed by incubation at 29 °C at 220 r/min for 96 h. During the fermentation process, 2 mL samples were collected at 24 h, 48 h, 72 h and 96 h respectively and transferred to sterile tubes for subsequent assays. The OD_600_ values were measured at each time interval to evaluate the growth profile. After 96 h of fermentation, the entire culture broth in each flask was transferred to 50 mL centrifuge tubes and centrifuged at 4500 rpm at 4 °C for 10 min. The supernatant was carefully harvested and preserved at 4 °C for further analysis. To investigate the growth and metabolic stress of recombinant CENPK2, the OD_600_ values and oxygen species (ROS) production were measured to evaluate cell growth. Briefly, growth curves were plotted based on OD_600_ readings collected at different time points. DCFH-DA (Beyotime, Shanghai, China, #S0033) was used to assess intracellular ROS levels. Briefly, CENPK2 cells were seeded into black-walled 96-well plates at a density of 1 × 10^6–7^ cells/well. Cells were stained with 10μM DCFH-DA in 0.9% NaCl for 20 min at 37 °C. PBS was used to wash the cells three times. Then, fluorescence was measured at 485 nm excitation/538 nm emission.

### 2.5. Tris-Tricine-SDS-PAGE Analysis

For the fermentation supernatant treatment, the collected samples were centrifuged, and the supernatant was 10-fold concentrated using a 3 kDa ultrafiltration membrane prior to electrophoresis.

Protein samples were separated by Tris-Tricine sodium dodecyl sulfate–polyacrylamide gel electrophoresis (Tris-Tricine-SDS-PAGE) according to the method of Schägger and von Jagow (1987). Tris-Tricine discontinuous gels consisted of a 15.5% resolving gel, a 10% spacer gel and a 4% stacking gel. Equal amounts of protein (12 μg) were loaded per lane. Electrophoresis was carried out at a constant voltage of 30 V for stacking gel and 100 V for separating gel until the dye front reached the bottom of the gel. Gels were then stained with ExBlue rapid protein dye for 30 min and washed with deionized water to remove floating color.

### 2.6. High-Performance Liquid Chromatography (HPLC) and Identification by Liquid Chromatography–Tandem Mass Spectrometry (LC-MS/MS)

HPLC analysis was carried out using a Thermo Fisher UltiMate 3000 HPLC system coupled with a UV detector (Waltham, MA, USA). Chromatographic separation was achieved using a C18 column (250 mm × 4.6 mm, 5 µm particle size). The detection wavelength was set to 220 nm. The mobile phase gradient was configured as follows: Solvent A: water containing 0.1% formic acid and Solvent B: acetonitrile containing 0.1% formic acid. The samples were loaded onto the trap column using the loading solvent at a flow rate of 3 µL/min from a temperature-controlled auto-sampler set at 30 °C. Loading was performed for 5 min before the sample was eluted onto the analytical column. The flow rate was set to 0.3 µL/min, and the gradient was generated as follows: 5% to 38% Solvent B from 0 to 22 min, 38% to 80% from 22 to 26 min, and 80% to 5% from 26 to 32 min using the Chromeleon non-linear gradient 5. Chromatography was performed at 30 °C.

The target peptide band was identified on the SDS-PAGE gel. The target band was excised with a sterile scalpel, and excess gel matrix was discarded, and the remaining gel block was minced into 1 mm^3^ particles. The gel particles were decolorized and dehydrated alternately with 25 mmol/L ammonium bicarbonate and acetonitrile (1:1, *v*/*v*) until fully transparent. Sequencing-grade trypsin (20 μg/mL, dissolved in 25 mmol/L ammonium bicarbonate) was added to the gel particles, and the mixture was incubated at 4 °C for 2 h. A small volume of 25 mmol/L ammonium bicarbonate was supplemented, followed by overnight digestion at 37 °C. After enzymatic digestion, the peptides were extracted with 5% (*v*/*v*) formic acid solution, vacuum-dried, and reconstituted in 0.1% (*v*/*v*) formic acid solution. The reconstituted peptide solution was filtered through a 0.22 μm membrane and submitted for analysis. Nano liquid chromatography–tandem mass spectrometry (nano LC-MS/MS) was performed by Sangon Biotech (Shanghai, China) Co., Ltd. Mass spectrometry parameters were set as follows: electrospray ionization (ESI) in positive ion mode; full-scan mass range: 300–1500 *m*/*z*; tandem mass spectrometry was operated in data-dependent acquisition (DDA) mode, with the top 20 most intense precursor ions selected for fragmentation at a collision energy of 28 eV. Raw mass spectrometry data were processed by the service provider via Mascot version 2.8.5.1 searching against the UniProt database.

### 2.7. In Vitro Hemolytic Toxicity

In this study, an in vitro hemolysis assay was performed using rabbit red blood cells (rRBCs). A total of 66 1.5 mL centrifuge tubes (EP tubes) were prepared and divided into the following groups: Tubes 1–27 (Api137 diluted in normal saline at various concentrations) served as the pure peptide experimental group. Tubes 28–30 (fermentation broth normal saline replacement (FB-NSR) group of R-CENPK2) and Tubes 31–33 (FB-NSR group for fermentation broth of wild-type CENPK2 (W-CENPK2). These two sets constituted the fermentation broth experimental groups. Tubes 34–36 were used as the positive control group, and Tubes 37–39 as the negative control group. Tubes 40–66 (melittin (MEL) group) served as the experimental reference group. Subsequently, 2% rRBC suspension, 0.9% NaCl, 10% Triton X-100, and the corresponding test samples were added to each tube according to the protocol listed in Table 2. After thorough mixing, the tubes were immediately incubated at 37 ± 0.5 °C for 4 h, and the phenotypes of hemolysis and agglutination were recorded. After centrifugation, 100 μL of the supernatant from each tube was transferred to a 96-well cell culture plate, and the absorbance at 540 nm (OD_540_) was measured. All tests were performed in three independent biological replicates.

### 2.8. In Vitro Antibacterial Assay

The cultivation conditions of pathogenic bacteria used in this study were as follows: *E. coli* ATCC K88, *S. Typhimurium* ATCC 9027, *S. aureus* CICC 21664, and *A. veronii* ATCC 13124 were cultured in LB medium at 37 °C. *L. monocytogenes* ATCC 19114 was cultured in TSB medium at 37 °C.

Strains were cultured overnight in the corresponding medium at the appropriate temperature, then inoculated into fresh medium and cultured for 2–3 h until the OD_600_ reached approximately 1.0. The bacterial culture was diluted to an OD_600_ of 0.01 (100-fold dilution), 10 μL of which was transferred to a 96-well plate for culture, with three parallel experiments set up. The negative control was added with 190 μL of LB or TSB medium, and the positive control was supplemented with ampicillin. To the experimental group, 190 μL of fermentation supernatant from each of the four strains was added: CENPK2, CENPK2 + Ty1-2/2, CENPK2 + Ty1-2/2/3, and CENPK2 + Ty1-2/2/3/4. All groups were cultured in parallel with the negative and positive controls for 12 h, and the antibacterial activity was determined with a microplate reader after culture. The formula for calculating the antibacterial rate is as follows: Antibacterial rate (%) = [(Mean absorbance of negative control − Absorbance of experimental group)/Mean absorbance of negative control] × 100%.

The minimum inhibitory concentrations (MICs) and minimum bactericidal concentrations (MBCs) of all synthetic AMPs and control antibiotics were determined following the CLSI M07-A10 standard broth microdilution method ([15]) with minor modifications [16]. Briefly, clinical isolates including *E. coli* ATCC K88, *S. Typhimurium* ATCC 9027, *S. aureus* CICC 21664, and *A. veronii* ATCC 13124 were cultured in LB medium at 37 °C. *L. monocytogenes* ATCC 19114 was cultured in TSB medium at 37 °C. Single colonies were resuspended in LB or TSB medium and cultured until mid-logarithmic phase (OD_600_ ≈ 0.4–0.6). The bacterial suspension was further diluted at a 1:100 ratio to prepare a final inoculum concentration of 1–2 × 10^6^ CFU per milliliter for each well of the microplate.

Two-fold serial dilutions of Api137 stock solutions (dissolved in sterile deionized water) were prepared in 96-well round-bottom plates, with concentration gradients ranging from 1 μM to 128 μM, with 100 μL of peptide solution per well. An equal volume (100 μL) of aforementioned bacterial suspension was added into each well to reach a total volume of 100 μL. Sterile LB/TSB only served as the blank control, bacteria without peptide as the negative control, and bacteria with ampicillin as the positive control. Plates were incubated statically at 37 °C for 18 h. The MIC value was defined as the lowest peptide concentration with no visible turbidity.

For the MBC measurement, 50 μL liquid from each clear well at and above the MIC was transferred onto TSA agar plates and incubated at 37 °C for 18–24 h. MBC was defined as the lowest sample concentration that resulted in no visible colony growth on the agar plates. All tests were performed in three independent biological replicates.

### 2.9. Cytotoxicity Assay

293T cells and DMEM medium (containing serum and double antibodies) were placed in a TC-treated culture dish at a certain ratio and incubated in a carbon dioxide incubator (37 °C, 5% CO_2_) for 72 h, then diluted to 5 × 10^4^ cells/mL with DMEM medium (containing serum and double antibodies).

Frozen pure Api137 antimicrobial peptide was thoroughly dissolved in DMEM medium supplemented with serum and penicillin–streptomycin (double antibiotics) to prepare gradient solutions at concentrations of 256, 128, 64, 32, 16, 8 and 4 μM. Fermentation supernatants of R-CENPK2 and W-CENPK2 strains (harvested after 96 h cultivation) were subjected to buffer exchange using 3 kDa ultrafiltration tubes, replacing the original fermentation medium with the same serum- and antibiotic-supplemented DMEM.

Following 24 h of incubation, the 96-well plate was retrieved, the spent medium was discarded, and cells were washed twice with PBS. Next, 100 μL of the serially diluted peptide solutions and buffer-exchanged fermentation supernatants were added to designated wells as experimental groups. After 24 h incubation, 10 μL of CCK-8 detection reagent was added to each well, and the plate was incubated for 1 h in the cell culture incubator. Absorbance at 450 nm was subsequently measured using a microplate reader. All tests were performed in three independent biological replicates.

## 3. Results

### 3.1. Construction of Recombinant Strains of CENPK2

**Figure 1:**
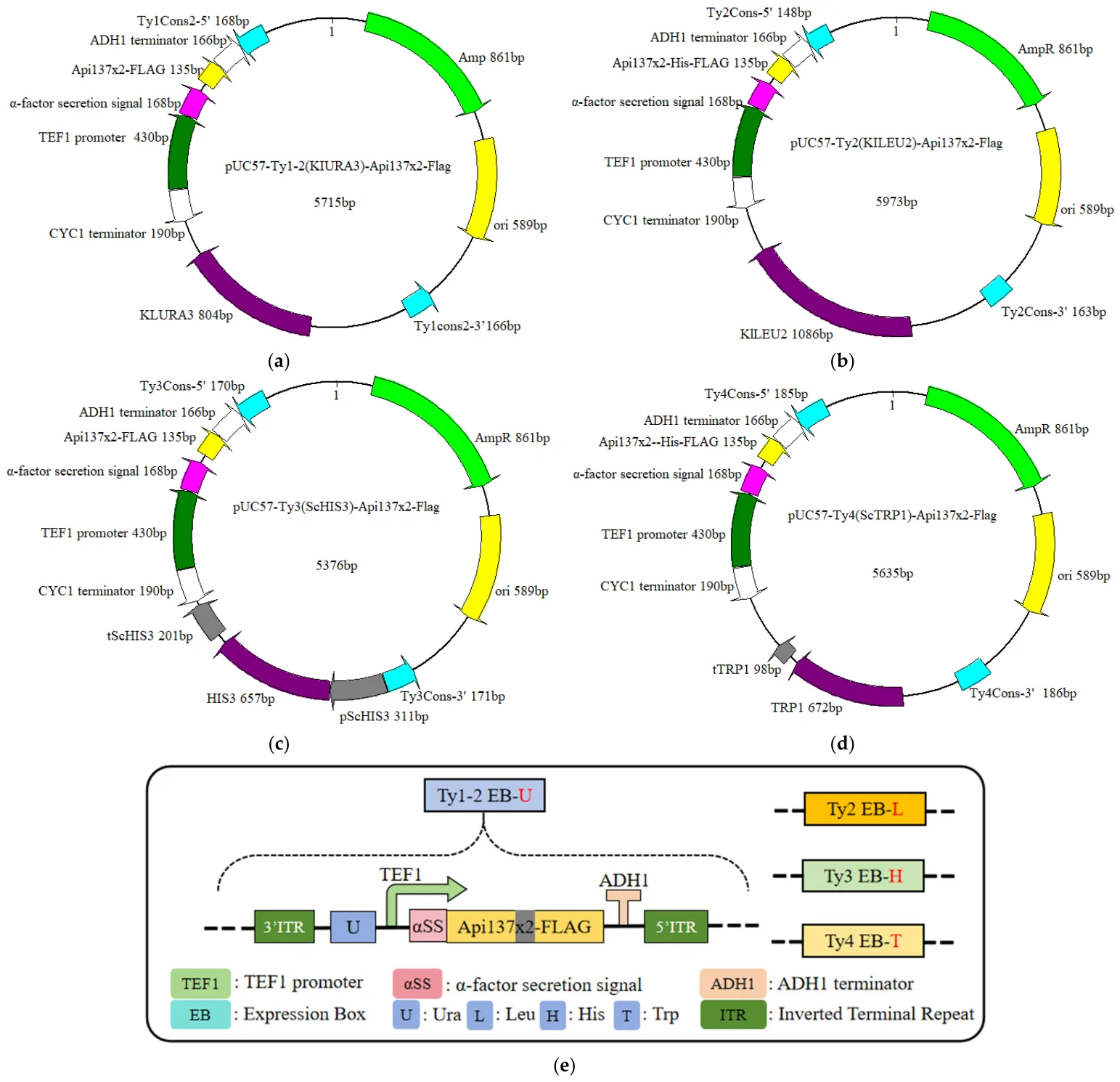
Map of plasmids: (**a**) (Ty1-2) pUC57-Ty1-2-KIURA3-Api137x2-FLAG, (**b**) (Ty2) pUC57-Ty2-KILEU2-Api137x2-FLAG, (**c**) (Ty3) pUC57-Ty3-ScHIS3-Api137x2-FLAG and (**d**) (Ty4) pUC57-Ty4-ScTRP1-Api137x2-FLAG. Corresponding screening tag (purple part) and an expression box of the target protein are flanked by 3′ITR and 5′ITR. (**e**) Schematic diagram of the integration and expression cassette for Api137x2-FLAG in *S. cerevisiae*. The construct is flanked by inverted terminal repeats (ITRs) of the Ty retrotransposon. The expression cassette contains the TEF1 promoter, the α-factor secretion signal (αSS), the Api137x2-FLAG coding sequence, and the ADH1 terminator. Selection markers include Ura (U), Leu (L), His (H), and Trp (T), each carried in independent expression boxes (EBs) designed for different Ty loci.

**Figure 2:**
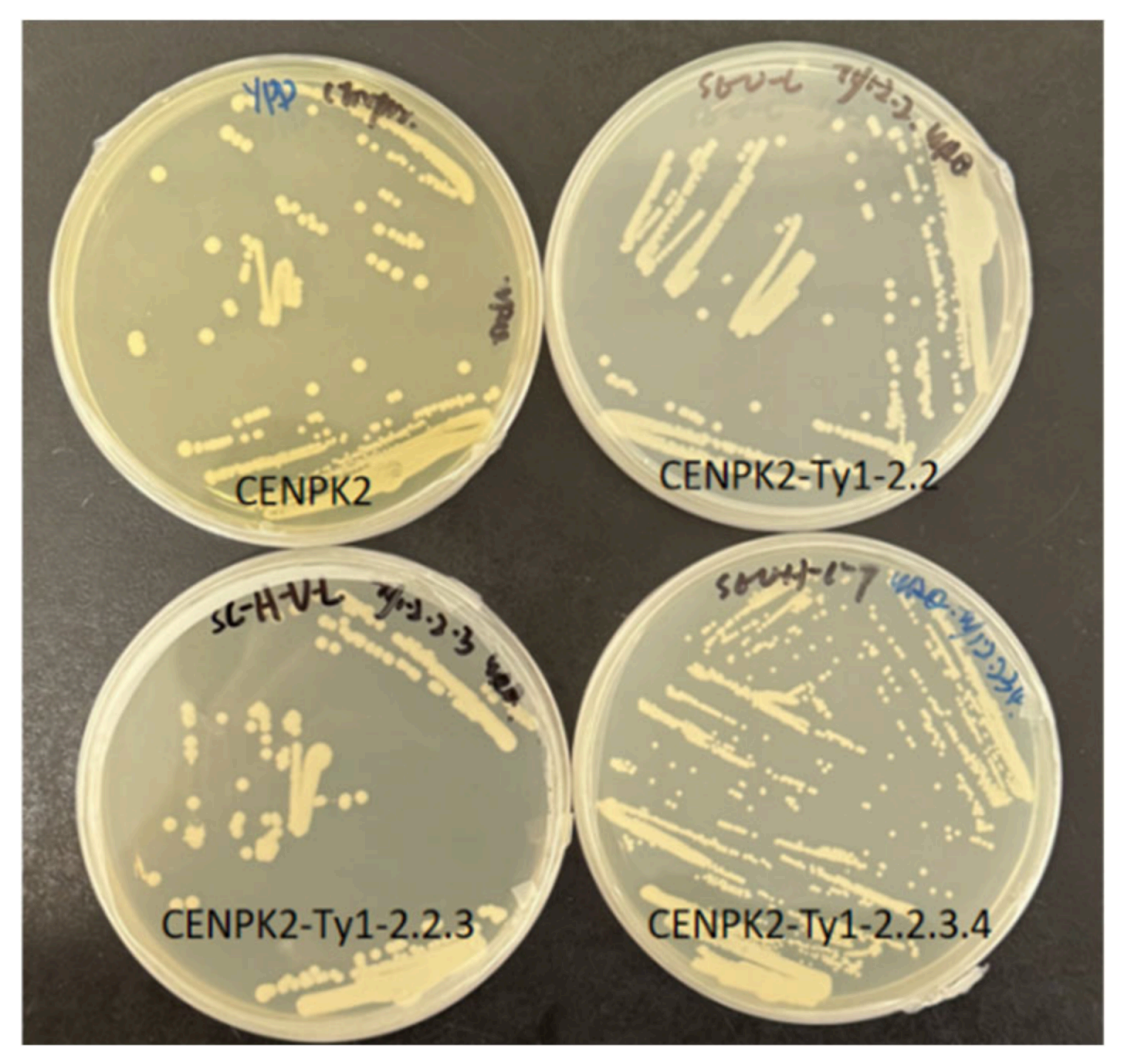
Screening and activity verification of electroporated CENPK2 on selective dropout media (YPD control: SC-U-L, SC-U-L-H, SC-U-L-H-T).

The maps and schematic diagrams of the Ty1-2, Ty2, Ty3 and Ty4 are shown in Figure 1. The target fragment from the constructed plasmid was introduced into CENPK2. Stable growth of transformants after multiple passages on selective medium confirmed successful insertion of the peptide expression cassette into the yeast genome, revealing stable maintenance of the integrated genetic element during cultivation (Figure 2).

### 3.2. Metabolic Burden of Recombinant Strains of CENPK2

**Figure 3:**
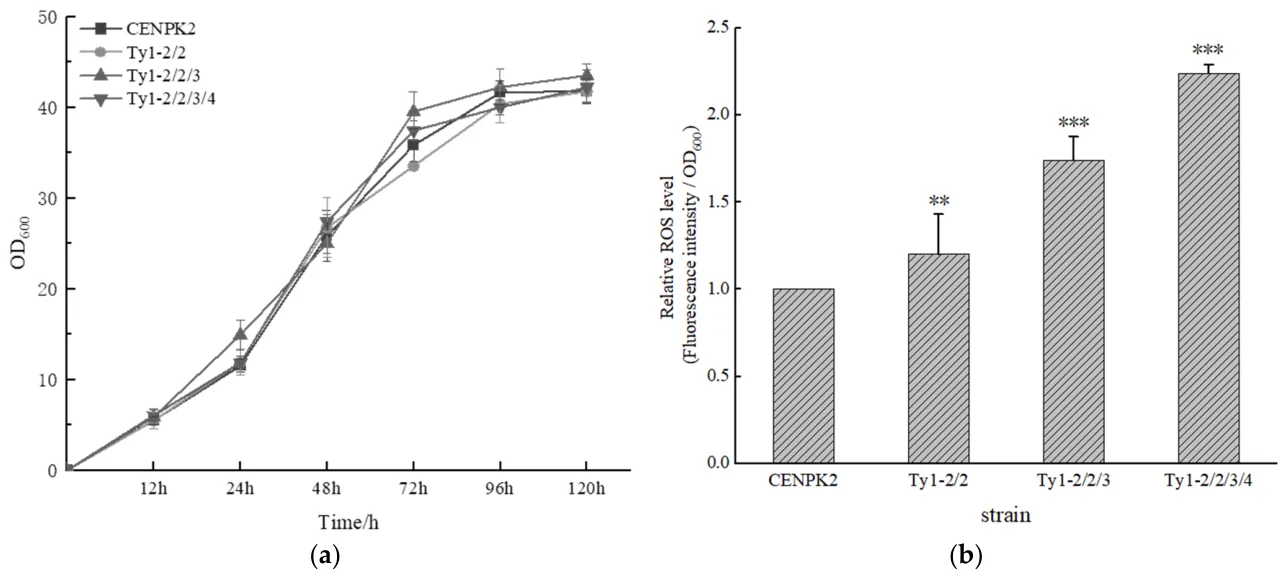
(**a**) Growth curves of the wild-type CENPK2 and the recombinant strains. (**b**) Intracellular ROS was detected with DCFH-DA fluorescent probe. Fluorescence intensity was normalized to OD_600_ to eliminate cell density bias. Relative ROS fold change was normalized to the average ROS level of CENPK2. One-way ANOVA with Dunnett’s post hoc test was applied for statistical analysis between each engineered strain and CENPK2. n ≥ 3; ** *p* < 0.01, *** *p* < 0.001.

To assess the impact of antimicrobial peptide expression on strain growth, we compared the growth profiles of the CENPK2 strain and its recombinant derivatives (Figure 3a). All strains exhibited comparable growth kinetics with no significant differences observed throughout the culture period, and maximum biomass accumulation was achieved at 120 h for all tested strains. The OD_600_ value of the CENPK2 strain was 41.79, whereas that of the CENPK2 + Ty1-2/2/3/4 strain reached 42.20. For ROS measurement using the DCFH-DA fluorescent probe assay (Figure 3b), the results revealed that ROS levels in the recombinant CENPK2 were slightly higher than those in the wild-type CENPK2, yet remained within a reasonable range. These results demonstrated that heterologous expression of the antimicrobial peptide Api137 exerted no obvious inhibitory or growth-promoting effects on the growth of CENPK2.

### 3.3. Tris-Tricine-SDS-PAGE Results of R-CENPK2

**Figure 4:**
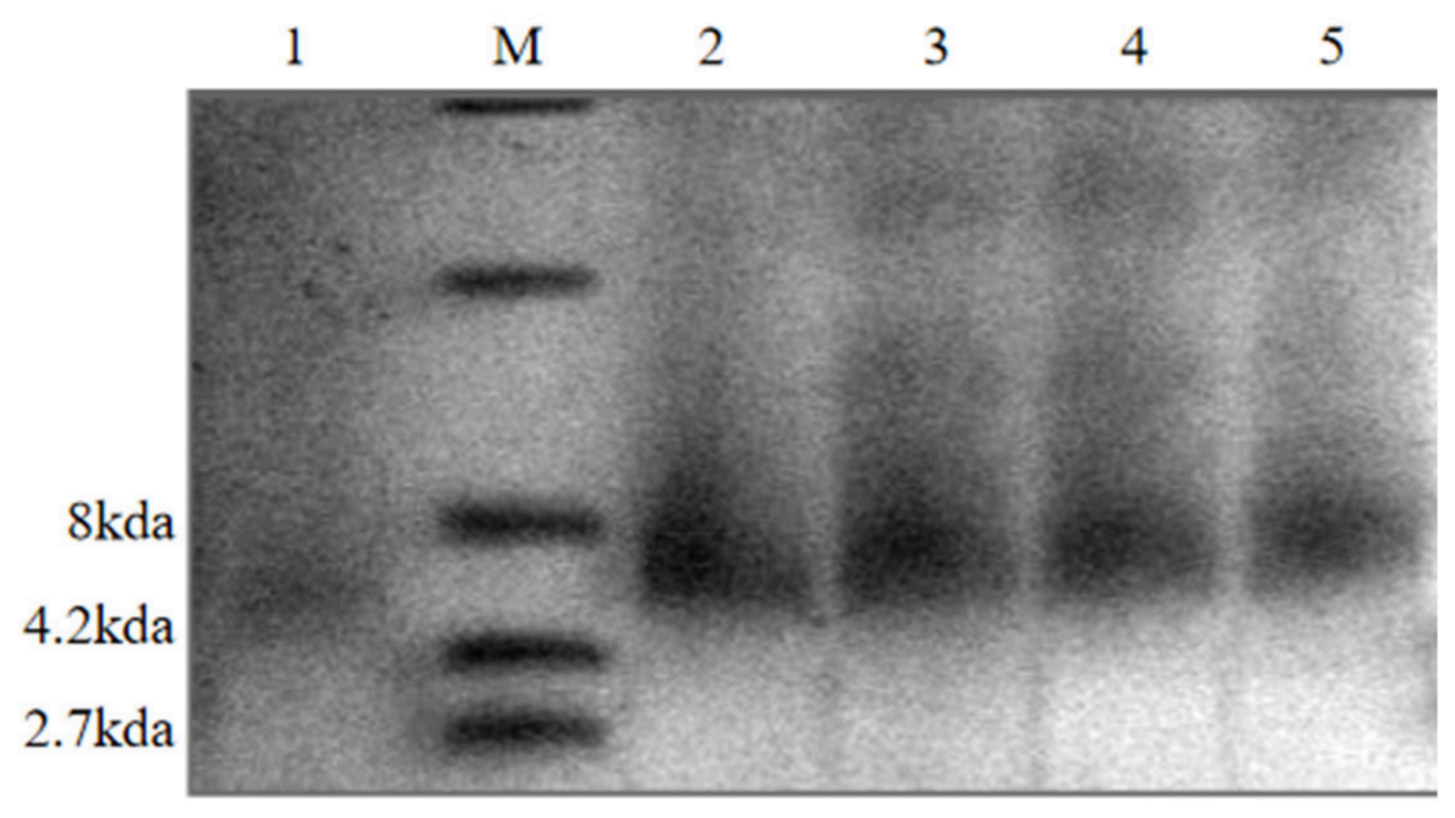
Verification of protein molecular weight. Lane M: 40 kDa protein ladder. Lane 1: fermentation supernatant of CENPK2 as blank. Lanes 2–5: fermentation supernatant of CENPK2 + Ty1-2/2/3/4 sampled at 96, 72, 48, and 24 h, respectively.

Tris-tricine-SDS-PAGE was employed to detect the target peptide using the fermentation broth of CENPK2 + Ty1-2/2/3/4. As shown in Figure 4, the target peptide encoded by the tandem Api137 expression cassette showed a molecular weight of 5.2 kDa. This observed molecular weight was consistent with the theoretical value, confirming that the R-CENPK2 strain was capable of expressing the target peptide stably.

### 3.4. High-Performance Liquid Chromatography (HPLC) and Identification Results of Liquid Chromatography–Tandem Mass Spectrometry (LC-MS/MS)

**Figure 5:**
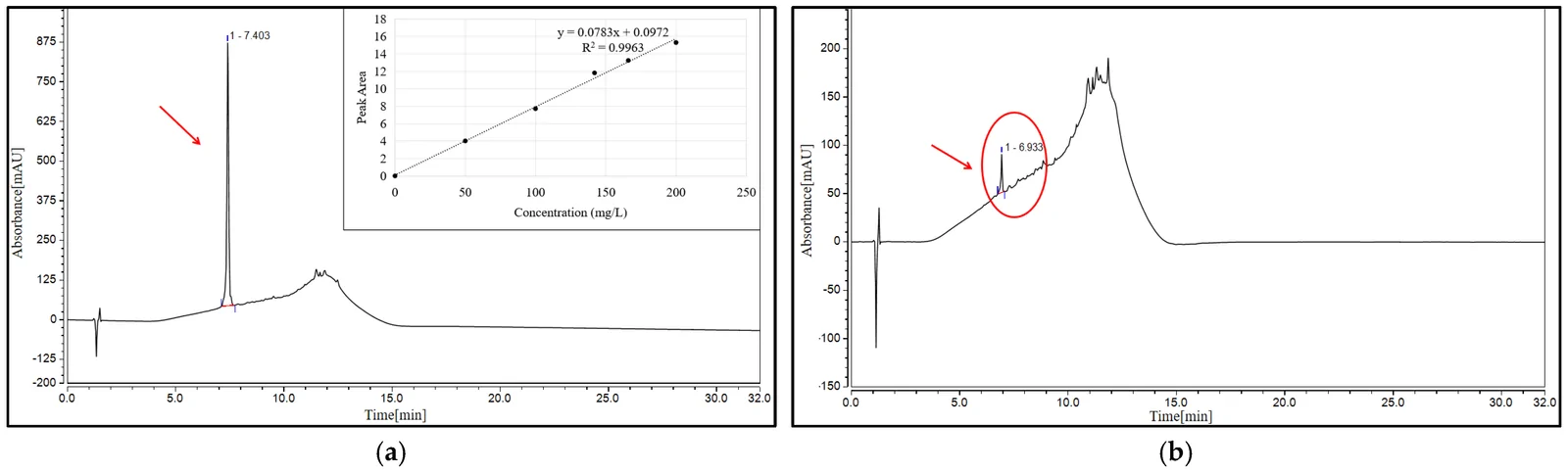

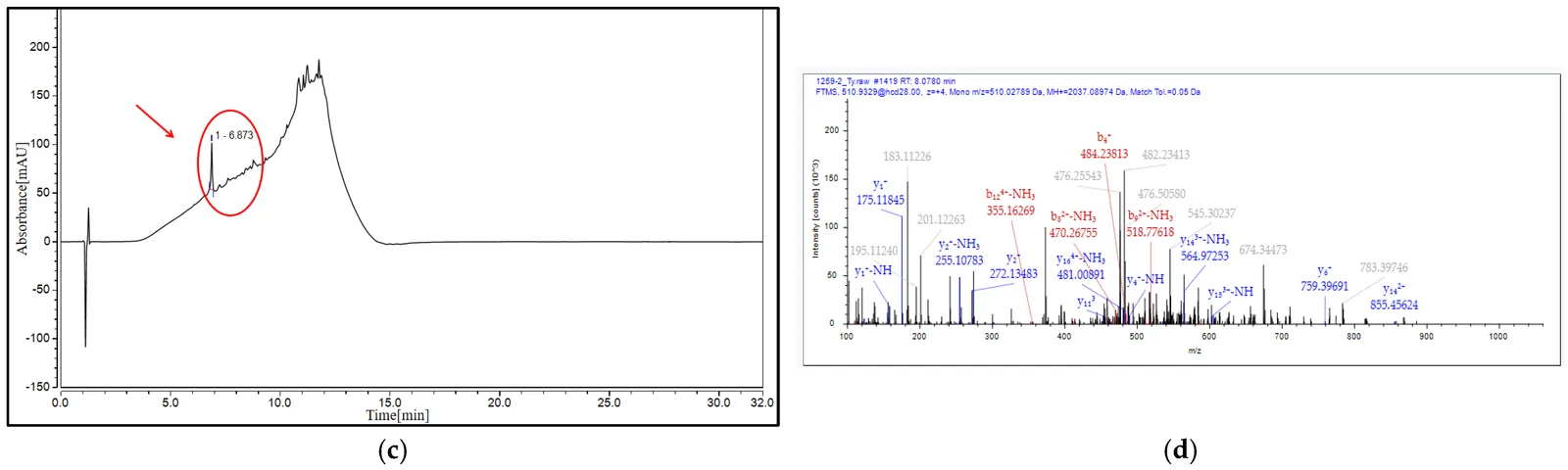
(**a**) Chromatograms of chemically synthesized Api137 standard and regression standard curve; linear regression was performed with standard concentration (0–200 mg/L) as the abscissa and chromatographic peak area as the ordinate. (**b**) Fermentation supernatant and (**c**) cell pellets. Retention time deviations were attributed to instrumental errors and trace differences in endogenous components between standards and fermentation samples. (**d**) ESI-MS/MS spectrum of the target peptide Tyrov (RT = 8.0780 min).

Recombinant enterokinase (P4237-1000U) was applied to cleave the tandem peptide of the fermentation broth, followed by concentration calculation according to the regression equation y = 0.0783x + 0.0972 with a correlation coefficient R2 = 0.9963, drawn by HPLC using chemically synthesized Api137. The standard curve indicated good linearity within the range of 0–200 mg/L. The results (Figure 5a–c) showed that the yield of the tandem peptide in the fermentation supernatant reached 20.5 mg/L. Owing to the presence of the α-factor secretion signal peptide gene, cell pellets collected from fermentation broth were lysed for intracellular detection, and the intracellular peptide concentration was determined to be 10.1 mg/L. Accordingly, the intracellular retention rate of the recombinant yeast was 33.0%. It should be noted that after hydrolysis by enterokinase, two peptide segments were both obtained, one of which contained a Flag tag. We used the peptide without the Flag tag for quantification, so the peptide concentration measured here is slightly less than that of the tandem peptide segment. All detection samples were performed in three independent replicates, and the obtained data is the average of the sample group data.

In the LC-MS/MS analysis, the component eluting at a retention time of 8.0780 min (Figure 5d) displayed a dominant quasi-molecular ion at *m*/*z* 510.02789, corresponding to the [M + 4H]^4+^ charge state. The calculated monoisotopic molecular weight of 1258.9329 Da was in excellent agreement with the theoretical mass of the target peptide (1258.9329 Da), yielding a mass deviation of <0.05 Da.

Collision-induced dissociation (CID/HCD) of this precursor ion produced an MS/MS spectrum containing a series of diagnostic fragment ions: N-terminal b-series ions, including b_4_^+^ (*m*/*z* 484.23813), b_12_^2+^-NH_3_ (*m*/*z* 355.16269), and b_9_^2+^-NH_3_ (*m*/*z* 470.26755); C-terminal y-series ions, such as y_1_^+^-NH_3_ (*m*/*z* 195.11240), y_2_^2+^-NH_3_ (*m*/*z* 255.10783), and y_6_^+^ (*m*/*z* 759.39691).

Additionally, numerous metastable ions arising from neutral losses of NH_3_ (−17 Da) or H_2_O (−18 Da) (e.g., y_14_^+^-NH_3_, y_13_^+^-NH_3_) were observed, a hallmark of CID fragmentation. The complementary information provided by these b and y ion series established a robust foundation for de novo peptide sequencing and sequence validation.

### 3.5. Hemolysis Assay

**Figure 6:**
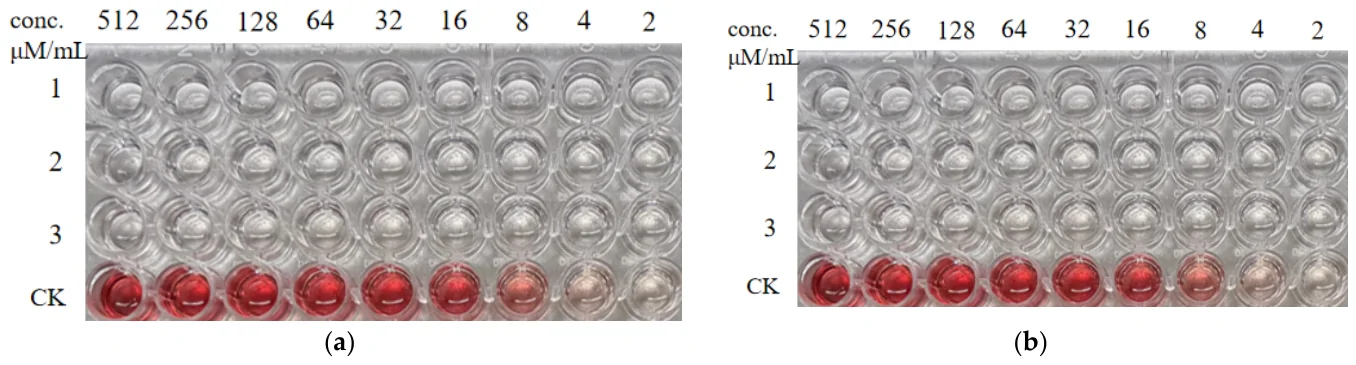
Physical state of the supernatant of each experimental group in a 96-well cell culture plate. (**a**) Physical state comparison between pure Api137 peptide and melittin; (**b**) physical state comparison between melittin FB-NSR solution of fermentation supernatant.

Following constant-temperature incubation, no hemolysis or agglutination was noted in negative control tubes, whereas complete hemolysis was observed in positive controls, validating the experimental system’s reliability and validity. Among the test groups, the supernatant of the MEL group appeared bright red, while those of the pure peptide and fermentation broth groups remained transparent (Figure 6).

Supernatant absorbance at 540 nm was measured using a microplate reader. Low heme levels were detected in Api137 peptide solutions (at various concentrations) and R/W-CENPK2 FB-NSR groups, which differed significantly from both control groups, confirming the absence of hemolysis in all experimental groups. The mean OD_540_ values were 1.213 for the positive control and 0.07 for the negative control.

**Figure 7:**
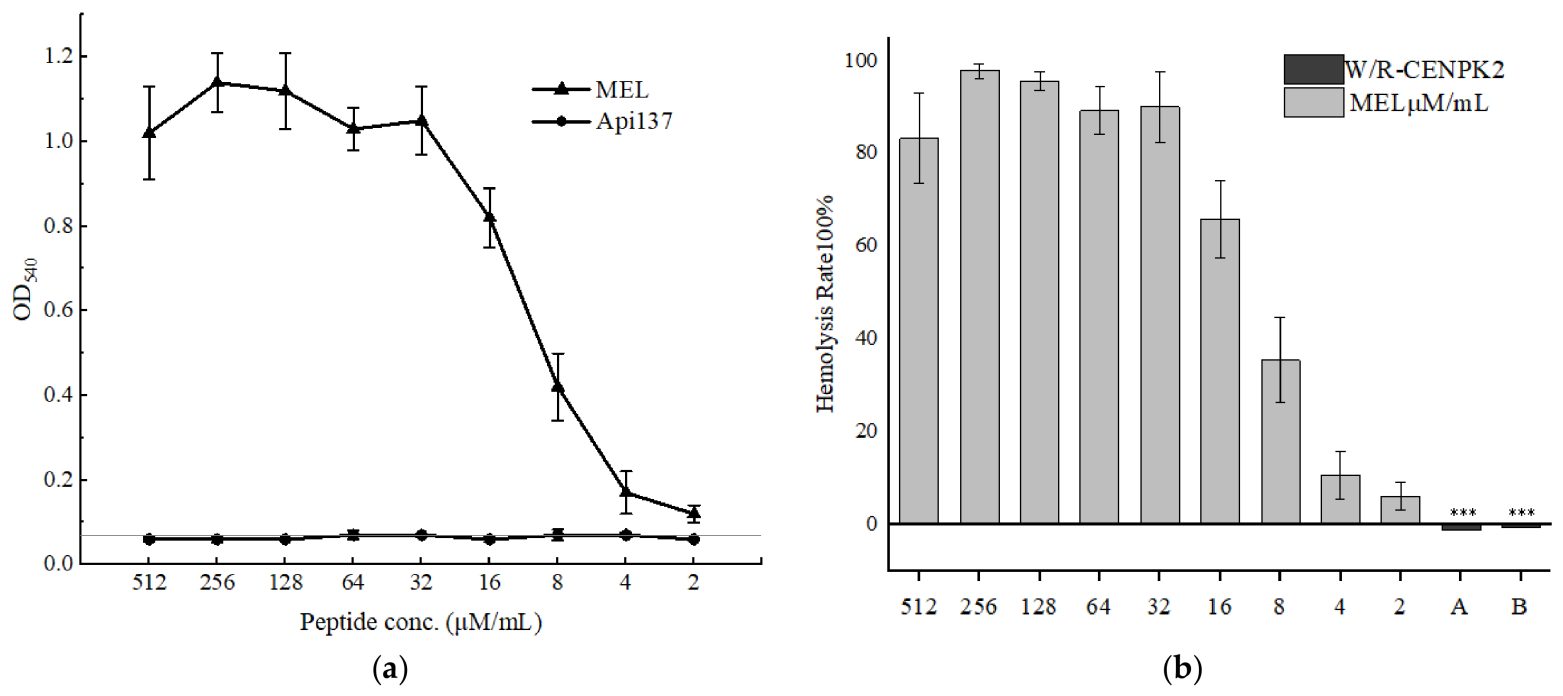
(**a**) Growth curves (OD_600_) of yeast treated with serially diluted MEL and purified Api137 peptide. The thin horizontal line denotes the OD_600_ value of the untreated negative control. Serially two-fold diluted peptide concentrations ranging from 512 μM to 2 μM were plotted on the *x*-axis. (**b**) Hemolytic activity of MEL and Api137 secreted by engineered W-CENPK2 and R-CENPK2 yeast strains. Hemolysis rate (%) was calculated by normalizing the absorbance of each treatment group to that of the 100% lysis positive control. Group A: supernatant of the W-CENPK2 strain, Group B: supernatant of the R-CENPK2 strain. Statistical comparisons between each sample group and the melittin positive control were performed via one-way ANOVA followed by Dunnett’s multiple comparison test. n ≥ 3; *** *p* < 0.001 denotes an extremely significant difference in hemolysis rate compared with the MEL group.

Curves comparing OD values (melittin vs. pure peptide groups) and hemolysis rates (MEL vs. fermentation broth groups) were generated (Figure 7). Minor negative hemolysis rates were observed due to instrument measurement errors: −0.4% for the W-CENPK2 group and −1.2% for the R-CENPK2 group, with several data points in the pure peptide group also falling below the negative reference line.

### 3.6. Antibacterial Activity Assessment

**Figure 8:**
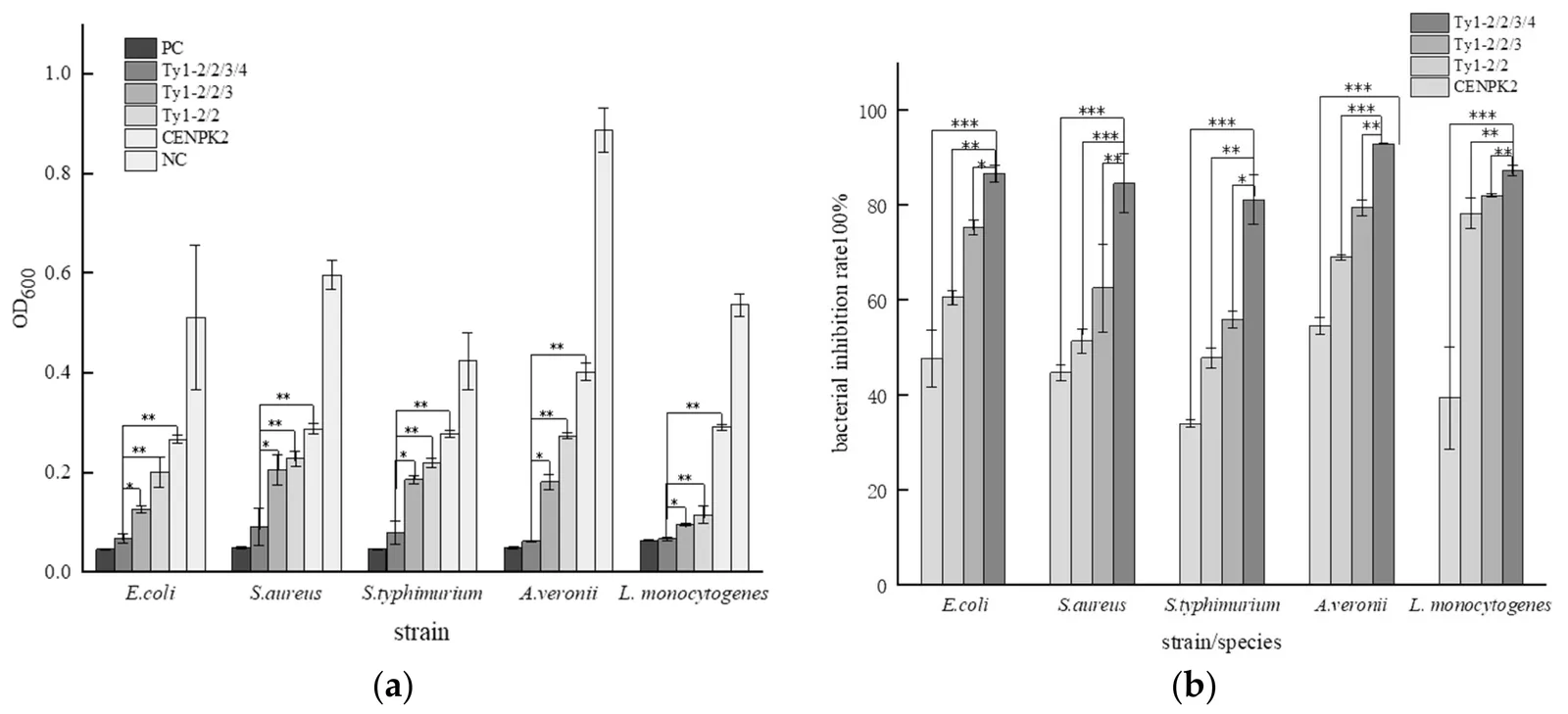
(**a**) OD_600_ values reflecting growth inhibition of *E. coli* ATCC K88, *S. Typhimurium* ATCC 9027, *S. aureus* CICC 21664, *A. veronii* ATCC 13124 and *L. monocytogenes* ATCC 19114 exposed to fermentation supernatants derived from distinct recombinant CENPK2 strains. PC, positive control (purified synthetic Api137 peptide); NC, negative control corresponding to wild-type CENPK2. (**b**) Relative antibacterial inhibition rates of fermentation supernatants from different recombinant CENPK2 strains against the tested pathogens. The antibacterial inhibition rate (%) was calculated by normalizing the OD_600_ value of each strain treatment group to the baseline value of the NC group. Statistical analysis was conducted via one-way ANOVA followed by Dunnett’s multiple comparison test, with each engineered strain compared against the (NC) group. n ≥ 3; * *p* < 0.05, ** *p* < 0.01, *** *p* < 0.001 denote significant differences relative to the NC group.

To assess the antibacterial activity of the antimicrobial peptide against the selected target bacteria, supernatants of CENPK2, CENPK2 + Ty1-2/2, CENPK2 + Ty1-2/2/3, and CENPK2 + Ty1-2/2/3/4 were co-cultured with the target bacteria. OD_600_ values of the experimental groups were determined to compute the respective antibacterial rates (Figure 8). The fermentation broth of CENPK2 + Ty1-2/2/3/4 exerted antibacterial activity against all tested target bacteria. Since unmodified CENPK2 inherently exhibited antibacterial activity, its fermentation broth was included in the assay to enhance experimental rigor. Among all strains, the CENPK2 + Ty1-2/2/3/4 fermentation broth exerted the strongest inhibitory effect on *A. veronii* ATCC 13124, with an average antibacterial rate of 92.9%, demonstrating effective suppression of target pathogenic bacterial proliferation.

Table 3 summarized the MIC and MBC profiles of the peptide Api137 against all tested pathogens. Peptide Api137 exhibited potent broad-spectrum activity against these strains, showing MIC values of 4–8 μM against *E. coli* ATCC K88 and corresponding MBC values of 8–16 μM, giving MBC/MIC ratios of 1 or 2. For *A. veronii* ATCC 13124, the MIC range was 8–16 μM and the MBC range was 32–64 μM, indicating strong bactericidal activity. In contrast, Api137 showed negligible activity against the *S. aureus* CICC 21664 and *L. monocytogenes* ATCC 19114, with MIC > 128 μM and undetectable MBC at the maximum tested concentration. Comparative analysis of MIC and MBC values revealed that the fermentatively produced recombinant Api137 exhibited slightly superior antibacterial activity relative to the chemically synthesized Api137.

### 3.7. Cytotoxicity Assessment Results

**Figure 9:**
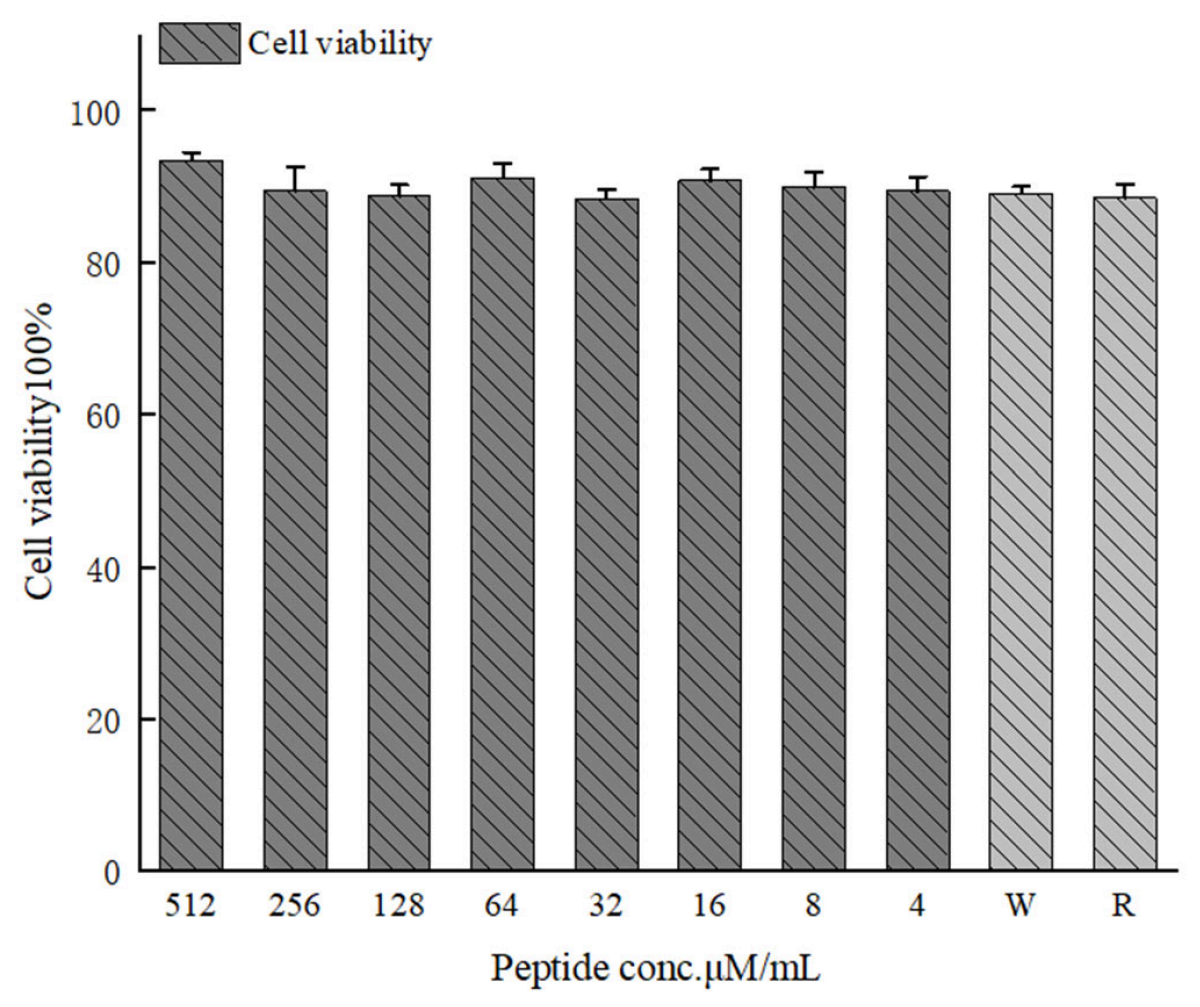
Cytotoxicity assay evaluating mammalian cell viability after treatment with serially diluted pure Api137 peptide (512–4 μM/mL) and fermentation supernatants from W-CENPK2 (W) and R-CENPK2 (R) strains. Cell viability percentage was normalized to the untreated blank cell control group (set as 100% viability). Statistical comparisons were performed via one-way ANOVA followed by Dunnett’s multiple comparisons test, with all treatment groups compared to the blank control. No statistically significant differences were observed across all groups (*p* > 0.05, n = 10 biological replicates).

Cytotoxicity of the antimicrobial peptide Api137 was assessed by examining the effects of Api137 peptide solutions at different concentration gradients and R/W-CENPK2 fermentation supernatants on the proliferation of human 293T cells. As shown in Figure 9, no statistically significant differences in cell proliferation were observed between groups treated with varying peptide concentrations, buffer-exchanged fermentation supernatant groups, and the control (CK) group (*p* > 0.05, n = 10). All Api137 solutions at different concentration gradients and medium-exchanged fermentation supernatants exhibited a toxicity grade of 0 (Table 4), confirming the absence of obvious cytotoxicity toward 293T cells.

## 4. Discussion

In this study, we optimized the Ty transposon system (Ty1-2, Ty2, Ty3, and Ty4) and employed multi-locus integration technology to successfully achieve the heterologous high-level expression of the proline-rich cationic antimicrobial peptide Api137 in *S. cerevisiae* CENPK2. The antibacterial activity and biosafety of the fermentation broth containing the target peptide were systematically verified. These results not only provide a feasible technical route for the industrial production of Api137, but also further expand the application of the yeast Ty transposon-mediated heterologous expression system in the biosynthesis of antimicrobial peptides.

Native Api137 suffers from limitations such as low in vivo content and difficult extraction and purification [3], while chemically synthesized antimicrobial peptides are associated with high production costs and unstable biological activity, restricting their large-scale application [17]. The eukaryotic expression system of *S. cerevisiae* was adopted to enable correct folding and post-translational modification of the target peptide. Moreover, the fermentation broth after simple centrifugation can exert direct antibacterial activity, greatly reducing production and purification costs. For proline-rich antimicrobial peptides represented by Api137, compared with prokaryotic expression systems (e.g., *E. coli*) prone to inclusion body formation [5,6], eukaryotic systems better maintain structural stability and biological activity, consistent with reports that eukaryotic hosts are more suitable for expressing proline-rich antimicrobial peptides.

In the present study, through multi-site modification of Ty1-Ty4 transposon loci, an expression cassette of Flag-tagged Api137 was integrated into the genome of CENPK2, fundamentally avoiding the instability of expression caused by extrachromosomal replication. As a laboratory strain with a high content of Ty transposons [11], CENPK2 provides natural and numerous integration sites for the target gene. The constructed CENPK2 + Ty1-2/2/3/4 showed no significant difference in growth curve compared with the CENPK2; ROS quantification assays revealed that although the intracellular ROS level of recombinant CENPK2 was slightly elevated, the accumulation remained within a physiologically safe threshold, indicating that multi-locus integration of Api137 did not impose an obvious metabolic burden on the host yeast, which is a key prerequisite for its industrial scale-up fermentation [7]. In vitro hemolysis assays showed that the fermentation broth of CENPK2 + Ty1-2/2/3/4 and pure Api137 peptide exhibited no hemolytic effect on rabbit erythrocytes (hemolysis rates of −1.2% for CENPK2 + Ty1-2/2/3/4 and −0.4% for CENPK2). CCK-8 cytotoxicity assays confirmed that the Api137 peptide and fermentation supernatant at different concentration gradients were classified as non-toxic to human 293T cells (*p* > 0.05). This is attributed to the specific targeting of Api137 to the 50S ribosomal subunit of prokaryotes, interfering with bacterial protein translation without cross-reactivity with eukaryotic ribosomes [5]. The excellent biosafety of the recombinant *S. cerevisiae* fermentation system renders Api137 a potential substitute for antibiotics in animal husbandry, effectively alleviating the problems of antibiotic abuse and the proliferation of drug-resistant bacteria [18,19].

In this study, a multi-dimensional verification strategy was adopted for the heterologous expression and activity of Api137. Tris-tricine-SDS-PAGE electrophoresis confirmed the expression of the target fusion protein consistent with the theoretical molecular weight (5.2 kDa). LC-MS/MS further verified the authenticity of the target peptide with a mass deviation of <0.05 Da, and its fragment ion information provided reliable evidence for the sequence identification of Api137. For the quantification, the extracellular yield of the tandem peptide in the fermentation supernatant reached 20.5 mg/L, and the intracellular retention rate of the recombinant CENPK2 strain was 33.0%. However, enterokinase specifically cleaves at the DDDK motif to excise the Flag tag. The tagged and mature peptides differ solely by the highly hydrophilic DYKDDDDK sequence, which results in only marginal differences in overall polarity. Furthermore, truncated peptide byproducts were formed in the fermentation process [20]. Incomplete digestion and slight non-specific cleavage adjacent to the DDDK cutting site also generate multiple truncated side products that differ from each other by only one or two amino acid residues [21,22]. These peptide variants exhibit nearly identical hydrophobic properties and thus co-elute under reversed-phase chromatographic conditions. Such co-elution may lead to peak overlap and precludes effective baseline separation; therefore, the peptide concentration measured is slightly less than that of actual value. A comparative analysis of MIC and MBC values revealed that the fermentatively produced recombinant Api137 exhibited slightly superior antibacterial activity relative to the chemically synthesized Api137. In vitro antibacterial experiments were performed on five common pathogenic bacteria (*E. coli* ATCC K88, *S. Typhimurium* ATCC 9027, *S. aureus* CICC 21664, *A. veronii* ATCC 13124 and *L. monocytogenes* ATCC 19114), and the crude yeast fermentation supernatant contains complex endogenous metabolites and native secreted components with potential intrinsic antibacterial activity. The wild-type fermentation supernatant reflects the baseline antibacterial activity contributed by intrinsic yeast metabolites, organic acids, native secretory proteins and other fermentation components with the fermentation broth of W-CENPK2 set as the control to eliminate interference from the host itself on the antibacterial results, rendering the inhibition rate data (up to 92.9% against *A. veronii* ATCC 13124) more accurate and credible. However, one critical observation deserves attention: the crude fermentation supernatant possessed certain antibacterial activity against Gram-positive pathogens, yet the chemically synthesized or cleaved monomeric Api137 peptide alone failed to produce obvious antibacterial effects against the same strains. We hypothesize that organic acids and other small-molecule compounds present in the fermentation supernatant may act synergistically with Api137 to potentiate the overall antimicrobial activity. However, given the absence of direct experimental evidence in the current study, this interpretation remains speculative and should be regarded as a working hypothesis rather than a confirmed mechanism. Elucidation of the underlying mechanistic basis will be pursued in our future investigations to validate this potential. Furthermore, the configuration of recombinants with different Ty transposon integration combinations (CENPK2 + Ty1-2/2, CENPK2 + Ty1-2/2/3, CENPK2 + Ty1-2/2/3/4) clarified the correlation between the number of integration sites and the antibacterial activity of the target peptide. It should be noted that, in the present study, the primary objective was to obtain the recombinant strain harboring the maximum number of integration loci while exhibiting optimal antibacterial activity. The intermediate strains were therefore included solely as references for comparative assessment of metabolic burden and antibacterial performance. Consequently, quantitative determination of Api137 production was performed exclusively for the quadruple-integrated strain, and no corresponding expression analysis was conducted for the intermediate constructs. As a result, the inference that an increased number of Ty integration sites correlates with elevated Api137 expression remains indirect. Definitive elucidation of this relationship would require additional quantitative measurements across strains with varying integration loci, which we have acknowledged as an important direction for future investigation.

Nevertheless, the present study also has certain drawbacks and limitations. The target antimicrobial peptide characterized in this study possesses an extremely low molecular weight with an ultra-short coding sequence. The limited sequence length prevents the design of nucleic acid probes with sufficient specificity and design of qPCR primers. Given this limitation, we only applied specific PCR and verified via selective culture medium screening to confirm successful insertion of exogenous cassettes into the yeast genome, and specific PCR can merely verify the occurrence of integration, but it cannot discriminate between single-copy and multi-copy insertions, nor realize quantitative measurement of integrated copy numbers. In addition, all fermentation experiments in this study were conducted in flasks with a fixed volume of 40 mL and a fixed fermentation time of 96 h. Flask fermentation suffers from drawbacks such as low oxygen transfer efficiency, uncontrollable pH and temperature, and unstable dissolved oxygen, failing to simulate actual industrial fermentation conditions [23]. Meanwhile, key fermentation parameters including carbon/nitrogen ratio, induction time, initial pH, and fermentation temperature were not optimized, limiting further improvement in Api137 expression yield. In addition, fermentation samples were collected only at 24 h, 48 h, 72 h, and 96 h, and the dynamic changes in Api137 expression level and antibacterial activity throughout the fermentation cycle were not investigated, which is unfavorable for determining the optimal harvest time. Although our research targets the livestock breeding application of engineered yeast strains, in practical industrial and breeding applications, whole fermentation broth (crude supernatant) rather than purified single peptide will be the final applied product. At present, Api137 in the fermentation supernatant is recovered via enterokinase-mediated cleavage of the Flag tag to release the monomeric peptide for quantitative detection. The concentration of the tandem peptide precursor was subsequently back-calculated from the quantified concentration of the monomeric peptide. The recombinant *S. cerevisiae* can efficiently express Api137, only ultrafiltration was used to concentrate the fermentation supernatant, and a simple and efficient purification process for Api137 was not established. Low purity of the target peptide and high purification costs would also affect its promotion in animal husbandry.

This study only verified the in vitro antibacterial activity, hemolytic properties, and cytotoxicity of Api137, without conducting in vivo experiments in animal infection models (e.g., *A. veronii* infection model). The in vivo efficacy, tissue distribution, metabolic pathway, and half-life of Api137 remain unclear [19]. In addition, fermentation broth was directly used in antibacterial assays, while pure Api137 peptide was only applied in cytotoxicity and hemolysis tests; the activity and safety of expressed Api137 in vivo still require further validation. For antimicrobial peptides, in vivo stability and bioavailability are key factors affecting their practical application, which have not been explored.

In this study, only five common pathogenic bacteria were selected for in vitro antibacterial assays, and solely the antibacterial activity of Api137 was investigated, without evaluating the synergistic antibacterial effects of Api137 combined with conventional antibiotics, other antimicrobial peptides, or preservatives. The combined use of multiple antibacterial agents can often reduce the application concentration of a single drug, delay the development of drug resistance, and enhance antibacterial efficacy [19], which represents an important research direction not addressed in the present work regarding the practical application of Api137.

To address the above shortcomings and limitations, the selection marker gene embedded in the expression cassette will be used as the target for qPCR primer design to resolve the problem of copy number quantitative analysis by optimizing the industrial fermentation process and additional strain modification to improve expression yield. On the basis of flask fermentation, fed-batch fermentation experiments can be conducted in bioreactors, and key fermentation parameters can be optimized via single-factor tests combined with response surface methodology to enhance the expression titer of Api137 [18]. On the basis of ultrafiltration concentration, chromatographic techniques can be integrated to establish a simple, efficient, and low-cost purification process for Api137, thereby obtaining a high-purity target peptide to meet application requirements in various fields including animal husbandry. Subsequently, animal infection models of different pathogenic bacteria (including clinical multidrug-resistant strains) will be constructed to verify the in vivo antibacterial efficacy of pure Api137 peptide and recombinant *S. cerevisiae* fermentation broth, and to evaluate the therapeutic dose, administration route, and biosafety of Api137 [5]. Meanwhile, pharmacokinetic studies will be performed to clarify the tissue distribution, metabolic pathway, half-life, and excretion pattern of Api137 in animals, providing a theoretical basis for formulating clinical and veterinary application regimens. In addition, long-term toxicity tests can be carried out to evaluate the potential side effects of prolonged Api137 administration, further ensuring its biosafety.

In summary, this study confirms that the Ty retrotransposon-mediated multi-locus integration strategy is an effective approach to achieve high-level heterologous expression of Api137 in *S. cerevisiae*. The constructed recombinant strain exhibits favorable characteristics including stable growth, high expression efficiency, excellent antibacterial activity, and favorable biosafety. Although this study has certain limitations in fermentation technology, in vivo validation, and purification procedures, it provides a solid experimental foundation and research direction for the industrial production and practical application of Api137. In the future, with the optimization of fermentation technology and the advancement of in vivo studies, Api137 is expected to serve as a highly efficient and safe alternative to antibiotics and be widely applied in animal husbandry, making a significant contribution to addressing the global problem of drug-resistant bacteria.

## 5. Conclusions

In this study, the Ty transposon system of the yeast expression plasmids was optimized, enabling CENPK2 to heterologously express the target peptide. Through multiple integration, the Ty loci were fully modified and utilized to obtain the CENPK2 + Ty1-2/2/3/4 containing four transposon genes. The results showed that the peptide from CENPK2 + Ty1-2/2/3/4 had significantly higher bioactivity and better antibacterial effect while ensuring safety by comparing the biological activities of the target peptides fermented by different CENPK2 strains. In summary, this experiment successfully expressed the antimicrobial peptide Api137 in *S. cerevisiae* and obtained a multifunctional peptide with higher bioactivity through Ty locus modification. The experimental results provided data support for the future application of the antimicrobial peptide Api137.

## Figures and Tables

**Table 1:** Primers used in the experiment.

Primer Name	Sequence (5′-3′)	Source
TEF1-F	TCGGTTAGAGCGGATGCATGAGTGATCCCCCACACACCATAG	Synthesized by Sangon Biotech
Api137-1′-R	CCTTGTAATCCAATCTTGGATGTGGTGGTCTAGG	
Api137-2′-R	TCATAAATCATAAGAAATTCGCGTCACAATCTTGGATGTGGTG	
Api137-2′-F	TCCAAGATTGGATTACAAGGATGATGACGAC	
ADH1-seq	GGGACCTAGACTTCAGGTTG	
CYC1-seq	CATCCAGTCCAACGAAAGAG	
M13-M4	GTTTTCCCAGTCACGACGTTG	
M13-RV	CAGGAAACAGCTATGACCATG	

**Table 2:** Components for hemolysis assay (unit: mL).

EP Tube Number	1–27	28–30	31–33	34–36	37–39	40–66
2% Red blood cell suspension/mL	0.1	0.1	0.1	0.1	0.1	0.1
0.9% Nacl/mL	-	-	-	-	0.1	-
10% Triton-X-100/mL	-	-	-	0.1	-	-
Test sample/mL	0.1	0.1	0.1	-	-	0.1

Note: “-” indicates no addition.

**Table 3:** Antibacterial MIC and MBC of chemically synthesized Api137 and biologically synthesized Api137 against five pathogens. Concentrations were tested by two-fold serial dilution, and data are presented as concentration ranges (μM/mL).

Strain	Chemically Synthesized Api137		Biologically Synthesized Api137	
	MIC	MBC	MIC	MBC
*E. coli*	8–16	16–32	4–8	8–16
*S. aureus*	>128	>128	128	>128
*A. veronii*	16–32	64–128	8–16	32–64
*S. typhimurium*	4–8	8–16	4–8	8–16
*L. monocytogenes*	>128	>128	>128	>128

**Table 4:** Results of cytotoxicity detection.

Peptide Solution (μM/mL)/Fermentation Broth	Absorbance Value	RGR (%)	Toxicity Grade
512	0.387 ± 0.0046	93.44 ± 1.1	0
256	0.372 ± 0.0131	89.46 ± 3.2	0
128	0.370 ± 0.0061	88.85 ± 1.5	0
64	0.376 ± 0.0081	91.17 ± 2.0	0
32	0.369 ± 0.0055	88.43 ± 1.3	0
16	0.375 ± 0.0060	90.87 ± 1.5	0
8	0.373 ± 0.0075	90.01 ± 1.9	0
4	0.371 ± 0.0078	89.46 ± 1.9	0
W-CENPK2	0.371 ± 0.0035	89.16 ± 0.9	0
R-CENPK2	0.369 ± 0.0067	88.60 ± 1.7	0

## Data Availability

The original contributions presented in this study are included in the article. Further inquiries can be directed to the corresponding authors.

## Abbreviations

The following abbreviations are used in this manuscript:

W-CENPK	Wild-type CENPK2
R-CENPK2	CENPK2 + Ty1-2/2/3/4
FB-NSR	Fermentation broth normal saline replacement
Ty1-2	pUC57-Ty1-2-KIURA3-Api137x2-FLAG
Ty2	pUC57-Ty2-KILEU2-Api137x2-FLAG
Ty3	pUC57-Ty3-ScHIS3-Api137x2-FLAG
Ty4	pUC57-Ty4-ScTRP1-Api137x2-FLAG

## References

[1] Magana M., Pushpanathan M., Santos A.L., Leanse L., Fernandez M., Ioannidis A., Giulianotti M.A., Apidianakis Y., Bradfute S., Ferguson A.L. (2020). The value of antimicrobial peptides in the age of resistance. Lancet Infect. Dis..

[2] Fusco A., Savio V., Donniacuo M., Perfetto B., Donnarumma G. (2021). Antimicrobial peptides human beta-defensin-2 and -3 protect the gut during candida albicans infections enhancing the intestinal barrier integrity: An in vitro study. Front. Cell. Infect. Microbiol..

[3] Zhang W.X., Tang X.Y., Zhang C.X., Zhao F.F., Zhang A.Z., Jiang N. (2025). Fusion expression and activity analysis of hybrid antimicrobial peptide CC28 and cellulase in Pichia pastoris. Chin. J. Anim. Husb..

[4] Knappe D., Schmidt R., Adermann K., Hoffmann R. (2019). Continuous subcutaneous delivery of proline-rich antimicrobial peptide Api137 provides superior efficacy to intravenous administration in a mouse infection model. Front. Microbiol..

[5] Lauer S.M., Gasse J., Krizsan A., Reepmeyer M., Sprink T., Nikolay R., Spahn C.M.T., Hoffmann R. (2025). The proline-rich antimicrobial peptide Api137 disrupts large ribosomal subunit assembly and induces misfolding. Nat. Commun..

[6] Reepmeyer M., Krizsan A., Brakel A., Kolano L., Gasse J., Husselbee B.W., Robinson A.J., Hoffmann R. (2025). Substitution of Proline Residues by 4-Fluoro-L-Proline Affects the Mechanism of the Proline-Rich Antimicrobial Peptide Api137. Antibiotics.

[7] Miao Z., Ren Y., Tarabini A., Yang L., Li H., Ye C., Liti G., Fischer G., Li J., Yue J.-X. (2025). ScRAPdb: An integrated pan-omics database for the Saccharomyces cerevisiae reference assembly panel. Nucleic Acids Res..

[8] Zhong K., Yan M.Z., Ruan S., Zhao R., Li J., Yang Y., Huang Z., Tang X. (2026). Effects of constructing Saccharomyces cerevisiae-Rhizopus oryzae fusant strain by protoplast fusion technology on ethanol metabolic pathway. Food Ferment. Ind..

[9] Qin L.Y., Sun X.H., Bin Q.L., Lei L.P., Zou H., Shan X.R., Lin S.Y., He D.D., Wang G.W., Wu R.Z. (2025). Research progress on modification of Saccharomyces cerevisiae by synthetic biology and gene manipulation technology. China Brew..

[10] Czaja W., Bensasson D., Ahn H.W., Garfinkel D.J., Bergman C.M. (2020). Evolution of Ty1 copy number control in yeast by horizontal transfer and recombination. PLoS Genet..

[11] Liti G., Carter D.M., Moses A.M., Warringer J., Parts L., James S.A., Davey R.P., Roberts I.N., Burt A., Koufopanou V. (2009). Population genomics of domestic and wild yeasts. Nature.

[12] Maury J., Germann S.M., Jacobsen S.A.B., Jensen N.B., Kildegaard K.R., Herrgård M.J., Schneider K., Koza A., Forster J., Nielsen J. (2016). EasyCloneMulti: A Set of Vectors for Simultaneous and Multiple Genomic Integrations in Saccharomyces cerevisiae. PLoS ONE.

[13] Jordan I.K., McDonald J.F. (1999). Phylogenetic perspective reveals abundant Ty1/Ty2 hybrid elements in the Saccharomyces cerevisiae genome. Mol. Biol. Evol..

[14] Qi L., Sui Y., Tang X.X., McGinty R.J., Liang X.-Z., Dominska M., Zhang K., Mirkin S.M., Zheng D.-Q., Petes T.D. (2023). Shuffling the yeast genome using CRISPR/Cas9-generated DSBs that target the transposable Ty1 elements. PLoS Genet..

[15] (2015). Methods for Dilution Antimicrobial Susceptibility Tests for Bacteria That Grow Aerobically.

[16] Zhang C.Q., Fu L., Zhu Y., Chen Q., Chen Z., Chang Y.F., Luo L. (2024). Antimicrobial activity of novel symmetrical antimicrobial peptides centered on a hydrophilic motif against resistant clinical isolates: In vitro and in vivo analyses. Microbiol. Spectr..

[17] Zhao M., Ma J., Zhang L., Wang Y., Liu H. (2024). Engineering strategies for enhanced heterologous protein production by Saccharomyces cerevisiae. Microb. Cell Fact..

[18] Sowers A., Wang G., Xing M., Li B. (2023). Advances in Antimicrobial Peptide Discovery via Machine Learning and Delivery via Nanotechnology. Microorganisms.

[19] Polikanov Y.S., Aleksashin N.A., Beckert B., Wilson D.N. (2018). The Mechanisms of Action of Ribosome-Targeting Peptide Antibiotics. Front. Mol. Biosci..

[20] Yang Y., Qiu X., Li J. (2015). Optimization of expression conditions for a novel NZ2114-derived antimicrobial peptide-MP1102 under the control of the GAP promoter in Pichia pastoris X-33. BMC Microbiol..

[21] Choi S.I., Song H.W., Moon J.W., Seong B.L. (2001). Recombinant enterokinase light chain with affinity tag: Expression from Saccharomyces cerevisiae and its utilities in fusion protein technology. Biotechnol. Bioeng..

[22] Likhareva V.V., Mikhailova A.G., Rumsh L.D. (2002). Non-specific (model) peptide hydrolysis by enteropeptidase and its possible physiological role. Vopr. Meditsinskoi Khimii.

[23] Rahn A., Behnken S., Hertel C. (2023). Industrial production of antimicrobial peptides in microbial hosts-current status and perspectives. Biotechnol. Adv..

